# Gene encoding a deubiquitinating enzyme is mutated in artesunate- and chloroquine-resistant rodent malaria parasites§

**DOI:** 10.1111/j.1365-2958.2007.05753.x

**Published:** 2007-07-01

**Authors:** Paul Hunt, Ana Afonso, Alison Creasey, Richard Culleton, Amar Bir Singh Sidhu, John Logan, Stephanie G Valderramos, Iain McNae, Sandra Cheesman, Virgilio do Rosario, Richard Carter, David A Fidock, Pedro Cravo

**Affiliations:** 1 Institute for Immunology and Infection Research, School of Biological Sciences, University of Edinburgh Ashworth Laboratory, Kings Buildings, Edinburgh EH9 3JT, UK.; 2 Centro de Malaria e Outras Doenças Tropicais/IHMT/UEI Malaria Rua da Junqueira 96, 1349-008 Lisbon, Portugal.; 3 Department of Microbiology and Immunology, Albert Einstein College of Medicine Forchheimer 403, 1300 Morris Park Avenue, Bronx, NY 10461, USA.; 4 Institute for Structural Biology, School of Biological Sciences, University of Edinburgh Swann Building, Kings Buildings, Edinburgh EH9 3JR, UK.; 5 Centro de Malaria e Outras Doenças Tropicais/IHMT/UEI Biologia Molecular Rua da Junqueira 96, 1349-008 Lisbon, Portugal.

## Abstract

Artemisinin- and artesunate-resistant *Plasmodium chabaudi* mutants, AS-ART and AS-ATN, were previously selected from chloroquine-resistant clones AS-30CQ and AS-15CQ respectively. Now, a genetic cross between AS-ART and the artemisinin-sensitive clone AJ has been analysed by Linkage Group Selection. A genetic linkage group on chromosome 2 was selected under artemisinin treatment. Within this locus, we identified two different mutations in a gene encoding a deubiquitinating enzyme. A distinct mutation occurred in each of the clones AS-30CQ and AS-ATN, relative to their respective progenitors in the AS lineage. The mutations occurred independently in different clones under drug selection with chloroquine (high concentration) or artesunate. Each mutation maps to a critical residue in a homologous human deubiquitinating protein structure. Although one mutation could theoretically account for the resistance of AS-ATN to artemisinin derivates, the other cannot account solely for the resistance of AS-ART, relative to the responses of its sensitive progenitor AS-30CQ. Two lines of *Plasmodium falciparum* with decreased susceptibility to artemisinin were also selected. Their drug-response phenotype was not genetically stable. No mutations in the UBP-1 gene encoding the *P. falciparum* orthologue of the deubiquitinating enzyme were observed. The possible significance of these mutations in parasite responses to chloroquine or artemisinin is discussed.

## Introduction

Malaria is estimated to cause the death of over one million people annually, mainly children in Africa. Moreover, despite intensive research, the overall disease burden arising from infections by the malaria parasite *Plasmodium falciparum* is increasing. One major factor has been the emergence and spread of malaria parasites which are resistant to antimalarial drugs such as chloroquine or pyrimethamine/sulphadoxine. Consequently, many countries have now introduced artemisinin (ART) derivatives as their first-line therapy, in combination with other drugs (such as mefloquine, amodiaquine, piperaquine, pyrimethamine/sulphadoxine or lumefantrine) (World Health Organization, 2006). These artemisinin combination therapies (ACTs) present favourable pharmacokinetics and are thought to reduce the probability of mutations that underlie resistance and treatment failure emerging in parasite populations (White, 1999). Artemisinin has a short half-life but acts extremely quickly in reducing parasite densities and symptoms. The activation, mechanisms of action and targets of artemisinin derivatives have been vigorously investigated and debated (Olliaro *et al*., 2001; Meshnick, 2002; Krishna *et al*., 2006). For instance, the activation of the endoperoxide group might produce a carbon-centred radical either after activation by Fe(II) in reduced haem (Meshnick *et al*., 1991) or as a result of interactions with iron-sulphur proteins in the mitochondrial electron-transport chain (Li *et al*., 2005). Candidate targets have included haem itself (Robert *et al*., 2005) and the SERCA-type Ca^2+^-ATPase (Eckstein-Ludwig *et al*., 2003). There is also debate regarding the extent to which chloroquine or artemisinin derivatives aggravate the oxidative stress normally incurred by parasites, for example during haemoglobin digestion and subsequent haem processing (oxidation of Fe^2+^-protoporphyrin IX to Fe^3+^-protoporphyrin IX in the food vacuole, with a concomitant release of reactive oxygen species) (reviewed in Tilley *et al*., 2001; Becker *et al*., 2004).

**Fig. 1:**
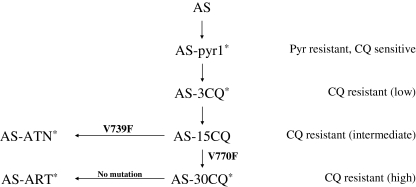
Drug-resistant mutants – the AS lineage. Mutants of *P. chabaudi* cloned isolate AS have been selected by passage in the presence of pyrimethamine, low, intermediate and high chloroquine concentrations, artesunate and artemisinin, to give lines and clones AS-Pyr (Walliker *et al*., 1975), AS-3CQ (Rosario, 1976; Carlton *et al*., 1998), AS-15CQ and AS-30CQ (Padua, 1981), AS-ATN and AS-ART (Afonso *et al*., 2006) respectively. An asterisk denotes that these clones have been used to generate genetic crosses with the cloned isolate, AJ. The genetic cross investigated in this study used AS-ART and AJ as parental clones. The mutations shown, V739F and V770F, refer to those in UBP-1, described in this study.

Afonso *et al*. (2006) recently reported the selection of two artemisinin-resistant mutant clones, AS-ART and AS-ATN (Fig. 1) in the rodent malaria parasite *Plasmodium chabaudi*. They were derived from chloroquine-resistant clones by serial passage in the presence of increasing but subcurative doses of artemisinin or artesunate (ATN) respectively. After cloning, both showed improved growth in the presence of either ART or ATN, relative to their sensitive progenitors, AS-30CQ and AS-15CQ. Minimum curative doses of artemisinin increased 15-fold in AS-ART and 26-fold in AS-ATN (relative to their sensitive progenitors) while those of artesunate increased five- to sixfold in both clones. These lines exhibited stable drug response phenotypes after cloning, passage in untreated mice and transmission through the mosquito vector.

What resistance mechanisms are possible and what are their genetic bases? Initial sequence analysis of these clones (and their sensitive progenitors) began with genes for which published evidence suggests a potential involvement in modulating parasite responses to artemisinin derivatives. These genes were the *P. chabaudi* orthologues of *pfatp6*, encoding the SERCA-type Ca^2+^-ATPase (Eckstein-Ludwig *et al*., 2003; Jambou *et al*., 2005; Uhlemann *et al*., 2005), *pfcrt* (Sidhu *et al*., 2002), *pfmdr1* (Reed *et al*., 2000; Ferrer-Rodríguez *et al*., 2004; Price *et al*., 2004; Sidhu *et al*., 2005) and *pftctp* (encoding translationally controlled tumour protein, TCTP) (Bhisutthibhan *et al*., 1998; Walker *et al*., 2000). Sequencing of AS-ART and AS-ATN and their progenitors (AS-30CQ and AS-15CQ) showed that there were no mutations or copy number changes in these genes (Afonso *et al*., 2006).

*Plasmodium chabaudi* is a robust model system for identifying genetic loci, candidate genes and individual mutations underlying drug resistance (Carlton *et al*., 2001). Classical genetic studies such as traditional linkage analysis (Carlton *et al*., 1998; Hayton *et al*., 2002; Cravo *et al*., 2003; Hunt *et al*., 2004a,b) or Linkage Group Selection (LGS) (Culleton *et al*., 2005), both of which analyse linkage between genotype and phenotype (Carter *et al*., 2007), have been employed to this end. LGS, used in this current investigation, characterizes the uncloned progeny of a genetic cross (between a resistant and sensitive parasite) by measuring the proportion of parental polymorphic markers at genome-wide loci, before and after drug treatment. Markers from the sensitive parent that are linked to the gene underlying the resistance phenotype are reduced in proportion or intensity after drug treatment, forming a ‘selection valley’. We have previously used amplified fragment length polymorphisms (AFLP) that distinguish the two parental lines in the genetic cross (Culleton *et al*., 2005; Martinelli *et al*., 2005a). A genetic linkage map (Martinelli *et al*., 2005b), a genome sequence database and a complete syntenic map (Kooij *et al*., 2006) allow us to map genes and markers genetically and physically, thus allowing identification of loci under selection. Mutations in candidate genes within the locus can be identified by comparative sequencing of genes from both the resistant mutant and its sensitive progenitor.

Here we describe the analysis of a genetic cross between AS-ART and AJ and the identification of a selection valley on *P. chabaudi* chromosome 2 detected by LGS analysis after artemisinin treatment. Subsequently we have identified two independent non-synonymous mutations in a gene encoding a deubiquitinating enzyme within this locus. One mutation appears in AS-ATN derived from AS-15CQ after artesunate selection and the other in AS-30CQ (also derived from AS-15CQ) after chloroquine selection.

## Results

### Genetic crosses and LGS

**Fig. 2:**
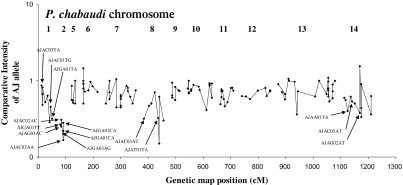
LGS – artemisin treatment of AS-ART × AJ genetic cross. The selection on a set of AJ-specific genome-wide AFLP markers (Grech *et al*., 2002) in the ‘treated’ group relative to the ‘untreated’ group is represented by Comparative Intensity (Martinelli *et al*., 2004) (logarithmic scale) of AFLP markers (vertical axis) and their position (revised, see Table 1) in a genetic linkage map (horizontal axis), assigned to specific chromosomes. AFLP markers mapped by sequence analysis (Fig. 3) are indicated.

We performed three independent genetic crosses between the artemisinin-resistant clone, AS-ART (Afonso *et al*., 2006) and the genetically distinct sensitive parasite clone AJ. Uncloned progeny from these crosses were pooled in equal proportions and passaged through two groups of mice. One group was treated with 25 mg artemisinin/kg/day for five consecutive days [day 0 to day 4 post infection (pi)]. The other group was untreated. Parasites were harvested on days 10 pi and 8 pi, respectively, and DNA samples from these groups were analysed by AFLP. The comparative intensities (CIs) for AFLP bands specific to the sensitive parent AJ, and their location within the *P. chabaudi* genetic linkage map are shown in Fig. 2.

**Fig. 3:**
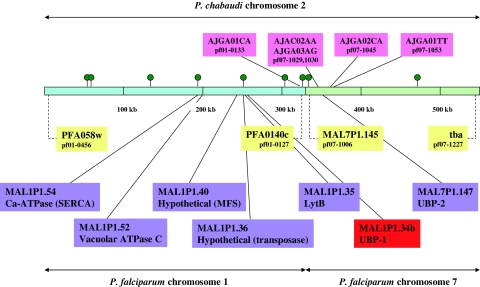
*P. chabaudi* chromosome 2 map – markers, genes and synteny. *P. chabaudi* chromosome 2 (blue + green bar; double-headed arrow, top) and its syntenic blocks relative to *P. falciparum* chromosome 1 (blue) and 7 (green) (double-headed arrows, bottom) are shown. The genes at the extremities of these blocks are shown with their chromosomal loci (pfxx-yyyy, where xx is the chromosome and yyyy the position in kb, yellow). Note that the syntenic block from *P. falciparum* chromosome 1 is reversed in direction. The position of AFLPs that were physically mapped (blast) and homologous positions in *P. falciparum* are shown (pink). Genes (not mutated) sequenced during current study are shown (purple). Mutated gene (UBP-1) is shown (red). The scale (100 kb intervals) indicates distances in *P. falciparum*. The positions of SNP allele quantification assays (pyrosequencing) are shown (green circles); these are, left to right, pf01–0406, pf01–0399, pf01–0323, pf01–0265, pf01–0197, pf01–0150, pf01–0129, pf07–1006 and pf07–1151.

**Table 1:** Physical and Genetic mapping of AFLP markers with low CI.

						Nearest gene				
						Nearest gene				
AFLP marker	Comparative intensity	*P. chabaudi* linkage group according to Martinelli *et al*. (2005b)	Contig ID	Length of contig (nt)	Approximate position of AFLP on contig	Gene	Gene annotation	Gene ID	*P. falciparum* genomic locus	*P. chabaudi* chromosome according to synteny Kooij *et al*. (2006)
AJGA01TA	0.32	Group 38	452.1	161 536	50 850	PFF0175c	Hypothetical	3885788	pf06–0144	1
AJAC01TG	0.45	Group 38	452.1	161 536	36 900	PFF0185c	Hypothetical	3885790	pf06–0160	1
AJAC03TA	0.48	Group 38	1290	95 398	92 500	PFF0480w	Hypothetical	3885877	pf06–0419	1
AJGA01CA	0.20	Chr 10	462	60 484	59 400	MAL1P1.71	Hypothetical	813167	pf01–0132	2
AJAC02AA	0.17	Chr 10	462	60 484	34 300	MAL7P1.147	Ubiquitin C-terminal hydrolase	2655023	pf07–1029	2
AJGA03AG	0.22	Chr 10	462	60 484	33 650	MAL7P1.147	Ubiquitin C-terminal hydrolase	2655023	pf07–1030	2
AJGA02CA	0.28	Chr 10	462	60 484	21 700	PF07_0108	Hypothetical	2655161	pf07–1045	2
AJAC02AC	0.31	Chr 10				Insufficient sequence for analysis			na	na
AJAG03AC	0.25	Chr 10				Insufficient sequence for analysis			na	na
AJGA01TT	0.27	Chr 10	462	60 484	12 810	MAL7P1.149	Hypothetical	2655024	pf07–1053	2
AJAC03AT	0.32	Chr 8	936.0	61 964	13 400	PFI0460w	Hypothetical	813372	pf09–0431	8
AJAT01TA	0.32	Chr 8	1489	7 907	2 150	PF1520w	Hypothetical	813584	pf09–1261	8
AJAG02AT	0.33	Group 2	408	63 353	12 000	PF13–0069	Translation initiation factor-2, putative	814047	pf13–0482	14
AJAC05AT	0.50	Group 2	1214	11 563	2 950	PFL0615w	Hypothetical	811176	pf12–0549	14
AJAA01TA	0.40	Group 2	882	61 223	49 600	PFL0805w	Hypothetical	811214	pf12–0665	14

The AJ-specific AFLP bands that show reduced CIs are shown with a physical location (*P. falciparum* genomic locus) of homologous positions in *P. falciparum* (pfxx-yyyy denotes locus yyyy kb along chromosome xx). This locus has been mapped to *P. chabaudi* chromosomes using detailed syntenic relationships (Kooij *et al*., 2006). The previously reported linkage groups (Martinelli *et al*., 2005b) are shown. Those markers previously allocated to ‘linkage group 38’ had not been reported by Martinelli *et al*. (2005b) because it contained less than eight markers. They have now been assigned to *P. falciparum* chromosome 1 on the basis of physical mapping described in the text. Similarly, markers previously allocated to ‘group 2’ have now been assigned to chromosome 14. Markers now assigned to chromosome 2 had previously been assigned (incorrectly) to chromosome 10. This assignment was based upon presumed linkage of these AFLP markers to a physically mapped RFLP marker, vacuolar ATPase subunit B (Karcz *et al*., 1994), for which the inheritance data were only available for nine (out of 28 possible) recombinant clones (Carlton *et al*., 1998). However, the inheritance data for another RFLP marker, Ca^2+^-dependent ATPase (Murakami *et al*., 1990), assigned by PFGE analysis to chromosome 2, were also only available for nine (out of 28 possible) clones (Carlton *et al*., 1998). Inspection of data showed that the group of AFLP markers, formerly assigned to chromosome 10 could also be linked to the Ca^2+^-dependent ATPase. Although this may be confirmed by characterization of the inheritance patterns of RFLPs Ca^2+^-dependent ATPase and vacuolar ATPase, subunit B, the data presented here demonstrates physical linking of the AFLP markers to chromosome 2.

Seven highly linked AFLP markers (mapping to chromosome 2) showed the lowest CIs. CIs of other AFLPs in three other linkage groups (mapping to chromosomes 1, 8 and 14) also appeared to be lower than those shown across the genome as a whole, suggesting possible selection or experimental variation at these loci.

### Mapping of AFLP markers

All of the AFLP bands showing reduced CIs had previously been placed on a genetic linkage map (Martinelli *et al*., 2005b). We confirmed the physical location of these AFLP markers (see *Experimental procedures*). The data for a number of AFLP markers in the four linkage groups under possible selection are shown in Table 1. Markers AJGA01TA, AJAC01TG and AJAC03TA all mapped to loci on *P. falciparum* chromosome 6 that are syntenic to *P. chabaudi* chromosome 1. AJGA01CA, AJAC02AA, AJGA02CA and AJGA01TT mapped to *P. falciparum* chromosome 1 or 7 at loci syntenic to *P. chabaudi* chromosome 2. AJAC03AT and AJAT01TA mapped to loci on *P. falciparum* chromosome 9 that are syntenic to *P. chabaudi* chromosome 8. Finally, AJAG02AT, AJAC05AT and AJAA01TA mapped to *P. falciparum* chromosome 13 or 12 at loci that are syntenic with *P. chabaudi* chromosome 14 (Table 1). Other markers were not mapped, either because of poor sequence quality or because marker sequence did not identify a *P. chabaudi* contig unambiguously.

*Plasmodium chabaudi* chromosome 2 is syntenic with two blocks of the *P. falciparum* genome, one each on chromosomes 1 and 7 (Kooij *et al*., 2006) (Fig. 3). These regions define approximately 551 kb of syntenic sequence in *P. falciparum*, encoding approximately 130 genes.

### Confirmation of selection by quantitative SNP analysis (pyrosequencing)

**Fig. 4:**
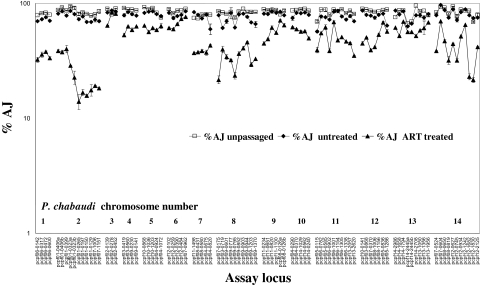
LGS – pyrosequencing analysis. The proportions of AJ alleles in the uncloned progeny prior to passage (‘unpassaged’), and after passage with (‘ART treated’) or without (‘untreated’) treatment with artemisinin are shown for 92 loci genome-wide. The loci are named (*x*-axis) after the positions of the *P. falciparum* orthologues using the same nomenclature as Table 1 and Fig. 3. Four loci (pf01–0406, pf01–0323, pf08–0126, pf14–2445) have two SNPs in the same assay and therefore give two sets of data, referred to as ‘a’ or ‘b’. Data shown are means, with standard errors indicated (for ‘treated’ and ‘untreated’, 3 < *n* < 8). Oligonucleotide primers for amplification and sequencing of loci are given in *Supplementary material*.

We wished to confirm independently the proportions of AJ alleles of genes in parasite populations after artemisinin treatment. We therefore measured proportions of AS and AJ SNPs at 93 loci distributed evenly across the genome, using pyrosequencing (Cheesman *et al*., 2007) which can measure the proportions of SNPs in a mixed population with greater precision and accuracy than proportional AFLP. The data (Fig. 4) show that the proportions of genome-wide AJ alleles after artemisinin treatment are generally reduced (relative to the untreated group) as expected, probably because of the loss of parental drug-sensitive AJ parasites during drug treatment. However, there were particularly large and consistent reductions at loci on chromosome 2 and, to a lesser extent, at loci on chromosomes 1, 8 and 14, giving further evidence of selection at these loci. Using pyrosequencing, we also estimated SNP proportions at loci on chromosomes 2, 8 and 14 in DNA derived from the three independent crosses between AS-ART and AJ. We observed reduced AJ proportions on chromosomes 2, 8 and 14, while loci on chromosome 6 (control) showed no selection in artemisinin-selected populations (*Supplementary material*).

### Identification of *ubp-1* mutation on *P. chabaudi* chromosome 2

**Fig. 5:**
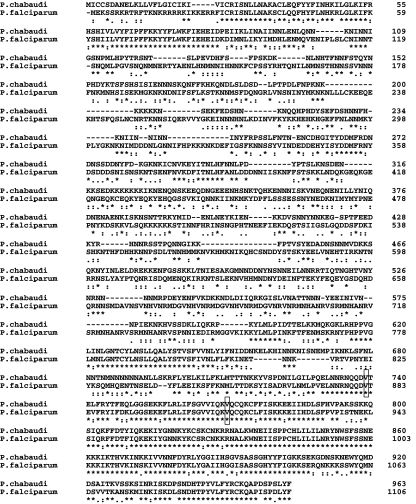
Alignment of *P. falciparum* (MAL1P1.34b) and its *P. chabaudi* orthologue UBP-1. *P. chabaudi* AS-30CQ mutated UBP-1 and the *P. falciparum* homologue MAL1P1.34b alignments are shown. The position of the mutation V770F (and the V739F mutation in AS-ATN) are indicated in rectangular boxes.

Within *P. chabaudi* chromosome 2, we sequenced a number of genes (Fig. 3) from AS-ART and AS-ATN to search for mutations that might account for selection of this locus during LGS. One gene, the orthologue of *P. falciparum* MAL1P1.34b, encodes a putative deubiquitinating protease (DUB), here termed ubiquitin-specific protease-1 (UBP-1). Within this gene, we identified two G to T mutations that are predicted to generate two non-synonymous substitutions in AS-ATN and AS-30CQ, respectively, namely V739F and V770F (Fig. 1). However, there was no mutation in AS-ART relative to AS-30CQ. Mutations that underlie the resistance of AS-ART to artemisinin (and presumably, artesunate), relative to those of AS-30CQ have therefore not been identified because the V770F mutation was already present in the AS-30CQ clone before ART selection. In order to evaluate the significance of the two mutations in the *P. chabaudi* homologue of MAL1P1.34b, especially regarding their possible role in *P. falciparum*, we aligned the predicted amino-acid sequences of the orthologous *P. falciparum* and the *P. chabaudi* proteins (Fig. 5). Extensive identity between the two sequences occurs close to the N-terminus and in a larger section in the C-terminus of the proteins. Both mutations map to the highly conserved C-terminal part of this DUB, suggesting that the mutations are located in functionally significant parts of the enzyme.

Because AS-ART markers on chromosome 2 appear to be positively selected by artemisinin treatment, we also sequenced, from AS-ART, another DUB homologue (orthologous to MAL71P.147) which also lies on the predicted locus of *P. chabaudi* chromosome 2 (Fig. 3). No mutation was identified in this gene (termed here as *ubp*-2) in the AS-ART clone. In order to discount the possibility that *ubp-1* mutations arose by chance during a burst of mutation activity around this locus (genomic region), we sequenced four other genes nearby, namely the *P. chabaudi* orthologues of MAL1.P1.35, MAL1P1.36, MAL1P1.40 and MAL1P1.52. None of these showed mutations in their predicted coding sequences or deviation from the *P. chabaudi* AS genome database entries.

### Mapping UBP-1 mutations in a crystal structure

**Fig. 6:**
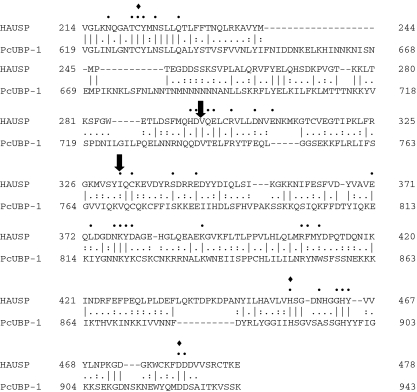
Alignment of *P. chabaudi* UBP-1 and human HAUSP. Alignment of human HAUSP (core protein) and *P. chabaudi* UBP-1. Residues marked ‘•’ are those with presumed catalytic function, role in substrate binding or making intramolecualar contacts which contribute to the structure (Hu *et al*., 2002). The three residues thought to be involved in catalysis are marked ‘♦’. The positions of mutations V739F and V770F are each indicated.

We used the highly conserved predicted C-terminal *P. chabaudi* amino acid sequence (aa 595–963) of UBP-1 to interrogate a protein structure database by blast search. We identified the core domain of a human homologue (herpes virus-associated ubiquitin-specific protease, HAUSP), for which the crystal structure has been solved (Hu *et al*., 2002). The alignment between the *P. chabaudi* UBP-1 and the HAUSP predicted protein sequences is shown (Fig. 6). Twenty-eight per cent of the *P. chabaudi* UBP-1 amino acids are identical to those of HAUSP, 23% are conservative substitutions and 49% are not conserved. We investigated whether those residues identified as having important structural roles (e.g. catalytic involvement, binding of ubiquitin) were particularly conserved between the two proteins. Out of 35 residues identified as having such roles (Hu *et al*., 2002) 25 (72%) were identical in the *P. chabaudi* UBP-1 sequence, five (14%) were conservative substitutions and only five (14%) residues showed non-conservative substitutions (Fig. 6). We therefore concluded that these homologues are likely to share many structural and functional similarities. The V739F mutation (equivalent to HAUSP V296) maps to an end of alpha helix 5 close to the proposed catalytic cysteine, C628 (equivalent to HAUSP C223). The other mutation, V770F (equivalent to HAUSP I332), maps to a hydrophobic pocket required for ubiquitin binding.

**Fig. 7:**
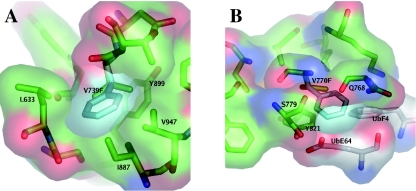
Structural consequences of UBP-1 mutations A. Residue environment around V739F. Side chains are shown as sticks with carbons coloured green for the *P. chabaudi* UBP-1 sequence. The mutation to F739 in the most likely orientation is represented in stick form with carbons coloured cyan. A surface representation is shown to indicate the tightness of fit around the phenylalanine. B. Residue environment around V770F. Side chains are shown as sticks with carbons coloured green for the *P. chabaudi* UBP-1 sequence and white for ubiquitin (UbF4, UbE64). The mutation to F770 in the most likely orientation is represented in stick form with carbons coloured cyan. A surface representation is shown to indicate the tightness of fit around the phenylalanine.

A homology model for the *P. chabaudi* UBP-1 amino acid sequence was produced using the catalytic core domain of HAUSP as a template. The model was checked manually and side-chain positions adjusted to minimize steric clashes. The wild-type *P. chabaudi* UBP-1 is likely to form a very similar structure to that found in HAUSP. For both the V739F and V770F mutations the large size increase of the side chain is likely to have a significant effect. In the HAUSP structure the valine 296 (V739F in *P. chabaudi*) side-chain points into a small hydrophobic pocket principally defined by Y465 and L454. In order for the V739F mutation to be accommodated, some movement of these side chains would be needed (Fig. 7A). It is most likely that the phenylalanine side chain would point into a small pocket produced by V947, I887 and L633. However, the V739F side chain comes too close to these residues to be accommodated without further movement. These movements are likely to affect the catalytic cysteine, C628, found on the same helix as L633. It is also likely that ubiquitin binding will be affected, because Y465 interacts directly with ubiquitin.

The V770F mutation also involves a large size increase in the side chain. The valine side-chain points into a small hydrophobic pocket that is principally defined by Y821 (UBP-1) and F4 (ubiquitin). The large increase in the size of the V770F mutation results in dramatic protein–protein clashes in side-chain orientations. A very small pocket is formed by Q768, S779 (UBP-1) and E64 (ubiquitin) and is shown in Fig. 7B. Even in this orientation the phenylalanine side chain comes too close to all these residues and a large amount of protein movement would be needed to accommodate it. It is therefore likely that the V770F mutation would disrupt ubiquitin binding in this region.

### Phenotypic characterization of ART-resistant *P. falciparum* lines

ART-resistant parasite lines of the NF54 and 7G8 genetic backgrounds were selected *in vitro* by exposing parasites to stepwise increases in ART concentrations. Selected drug-resistant lines were designated ART-R^NF54^ and ART-R^7G8^. The degree of ART resistance was determined by [^3^H]-hypoxanthine incorporation assays performed in parallel with their parental control lines. ART IC_50_ values for ART-R^NF54^ and ART-R^7G8^ were 148 and 98 nM, respectively, as compared with IC_50_ values of 30 and 20 nM for control lines NF54 and 7G8 respectively. These *in vitro* sensitivity assays showed that selection for resistance to ART resulted in approximately fivefold decrease in susceptibility to this drug in both resistant lines. To confirm the stability of the ART-resistant phenotype, frozen ART-R^NF54^ and ART-R^7G8^ parasite lines were thawed and after growing in the absence of drug pressure for 2 weeks, ART IC_50_ values were retested. These assays revealed ART IC_50_ values of 33 nM and 25 nM for ART-R^NF54^ and ART-R^7G8^ lines, respectively, which were comparable to their respective parental controls. This loss of the ART resistance phenotype in the drug-selected parasites indicates that ART-R^NF54^ and ART-R^7G8^ parasite lines acquired a transient drug-induced ART resistance phenotype.

### Sequence analysis of the *pfcrt, pfmdr1* and *pfubp-1* genes

To study the molecular basis of (transient) ART resistance, we polymerase chain reaction (PCR)-amplified and sequenced the *pfcrt, pfmdr1* and *pfubp-1* genes of ART-resistant and wild-type parasite lines. We selected these genetic determinants based on the observation that mutations in these genes modulate susceptibility to ART and other antimalarials. There were no sequence changes observed at the polymorphic positions of the *pfcrt* (position 76) and *pfmdr1* (positions 86, 1034, 1042 and 1246) genes of ART-R^NF54^ and ART-R^7G8^ lines grown in the presence of drug. Sequencing of the complete open reading frame of the *pfubp-1* gene of ART-resistant lines also did not reveal any sequence change compared with the parental lines, although two novel SNPs were detected in the 7G8 line at V260A and E265K. These investigations suggest that the transient resistance phenotye of the ART-R^NF54^ and ART-R^7G8^ lines was due to reversible modulation of gene expression.

## Discussion

We have used LGS to analyse the uncloned progeny of a genetic cross between the artemisinin- (and artesunate-) resistant clone, AS-ART and the sensitive clone AJ, before and after treatment with artemisinin, using AFLP marker intensities and genome-wide pyrosequencing. Using the pooled uncloned progeny from three independent genetic crosses, we obtained a selection valley on chromosome 2, and also evidence of selection on chromosomes 1, 8 and 14. An analysis of the three individual component genetic crosses by pyrosequencing (supplementary data) for loci on chromosomes 2, 6 (control), 8 and 14 confirms possible selection signatures on chromosome 2, 8 and 14. The similarity of our quantitative AFLP and pyrosequencing data confirms that genome-wide pyrosequencing is a useful tool for the *de novo* mapping of genes underlying selectable phenotypes in LGS-type experiments, as previously suggested (Cheesman *et al*., 2007).

Because *pcatp6* (encoding SERCA Ca^2+^-ATPase) is located within chromosome 2 (Fig. 3) and because its involvement in artemisinin activation and resistance has been proposed (Jambou *et al*., 2005; Uhlemann *et al*., 2005), we wished to investigate whether there were *pcatp6* mutations which might affect the activity or expression of this protein. However, there were no mutations in AS-ART coding region, over 4 kb of 5′-UT or over 1 kb of 3′-UT (Afonso *et al*., 2006).

Instead, within *P. chabaudi* chromosome 2, we identified two mutations in a gene encoding a DUB enzyme (UBP-1); V739F in the artesunate-resistant clone AS-ATN (relative to its progenitor AS-15CQ), and V770F in the high chloroquine-resistant clone AS-30CQ (relative to AS-15CQ). These two mutations within the same gene occurred independently in two different lines under selection with artesunate and chloroquine respectively. Each mutation produces an identical substitution (V to F), and occurs in one of only two regions that are highly conserved relative to the orthologous *P. falciparum* gene.

An analysis of the structure of a human homologue (HAUSP) of the mutated *P. chabaudi* protein, showed that residues underlying structural, intermolecular or catalytic roles in HAUSP are highly conserved in the *P. chabaudi* DUB. Both mutations increase the size of the side group and map onto residues that are likely to be critical to the molecule's activity.

Importantly, while it is theoretically possible to argue that V739F could confer artesunate/artemisinin resistance relative to AS-15CQ, there is no corresponding mutation to explain the resistance of AS-ART relative to AS-30CQ, because mutation V770F is present in both AS-ART and AS-30CQ.

Some reports have linked protein ubiquitination/deubiquitination to DNA repair function (Huang and D'Andrea, 2006). However, we reject the hypothesis that the mutations reported here may underlie a change in a phenotype known as Accelerated Resistance to Multiple Drugs, alternatively known as a ‘mutator’ phenotype (Rathod *et al*., 1997). Such mutations may occur during the original selection of drug-resistant mutants (i.e. at some stage during the generation of the drug-resistant AS lineage). However, these pre-existing mutations would not be selected during LGS experiments unless, of course, they are genetically linked to other, as yet, unidentified, mutations that underlie the resistance phenotype.

We have considered other possible interpretations. The first is simply that there is a mutation in a different gene (gene X) on chromosome 2 that arose during artemisinin selection on AS-30CQ during the AS-ART mutant generation. This mutation could be responsible for the selection valley obtained during LGS, and would differentiate AS-ART and AS-30CQ. Whether or not AS-ATN also has a mutation in the same (or similar) gene X relative to AS-15CQ is an open question that can be addressed by analysing a genetic cross between AS-ATN and AJ and by comparative sequencing of AS-ATN. We are currently investigating the presence of other mutations in this linkage group by further comparative sequencing of chromosome 2 genes from the resistant clones and their sensitive progenitors. If gene X is mutated in both AS-ATN and AS-ART, and if this mutation is causing the selection of chromosome 2 in LGS, what would be our interpretation of the UBP mutations reported here? One possibility is that UBP mutations, which are all relative to the parasite AS-15CQ, may compensate for mutations selected during the acquisition of intermediate resistance to CQ in AS-15CQ from AS-3CQ (resistant to low concentrations of chloroquine) (Fig. 1). This would suggest that a mutation in AS-15CQ has compromised the parasite's normal physiological fitness and that subsequent selections with other drugs have selected a compensatory UBP mutation that abrogates the fitness costs of the AS-15CQ mutation.

If, on the other hand, gene X lies on a different chromosome (for example, chromosomes 1, 8 and 14), we must consider why chromosome 2 shows a selection valley. We would suggest that the compensation of mutations underlying intermediate chloroquine resistance in AS-15CQ by mutations in UBP-1 is *required* for the expression of resistance to artemisinin, i.e. that the phenotypic expression of a mutation in gene X is epistatic to the UBP mutations.

We wish to emphasize one possible implication of this interpretation. The UBP-1 mutations that may fulfil this role occurred during artesunate selection of AS-ATN and during chloroquine selection of AS-30CQ. The fact that similar mutations in the same gene were selected during long-term passage in the presence of two different drugs suggests that there may be some underlying common cellular function that might be compromised during treatment with artemisinin derivatives or chloroquine, such as regulation of oxidative stress in drug-treated parasites (Krungkrai and Yuthavong, 1987; Tilley *et al*., 2001; Becker *et al*., 2004). Indeed a DUB enzyme was a primary target of oxidative stress in mutant superoxide dismutase-1 (SOD1) transgenic mice (Poon *et al*., 2005). Also, the expression of its homologue was modified by oxygen stress in a human tumour cell line; upregulation and downregulation were associated with surviving and apoptotic cells, respectively (Shen *et al*., 2006).

We also wish to suggest that the basis of artemisinin resistance is likely to be multigenic for the following reasons. Artemisinin resistance in *P. chabaudi* required significant passage using subcurative doses of artemisinin, and required the use of parasites with previously generated chloroquine resistance. Stable, artemisinin resistance is difficult to obtain with *P. falciparum* in culture. Selection of genetic crosses with artemisinin is suggesting an important contribution of a gene on chromosome 2, and perhaps, to a lesser extent, an involvement of other genes on, for example, chromosomes 1, 8 and 14.

We have also selected two lines of *P. falciparum* parasites that show decreased susceptibility to artemisinin but whose phenotype is not stable. These lines do not bear mutations in *pfubp-1*. In contrast, the *P. chabaudi* parasites AS-ART and AS-ATN are both genetically stable and selected *in vivo*. These factors emphasize the value of the *P. chabaudi* rodent malaria for the generation and genetic characterization of mutations underlying drug resistance. We await with interest a characterization of DUB enzymes in genetically stable artemisinin-resistant *P. falciparum* mutants.

What effect might UBP-1 mutations have on malarial parasites? We cautiously expect that a partial loss of UBP-1 activity might lead to an increase in degradation (via the proteasome) of proteins that act as UBP-1 substrates. Identification of UBP-1 substrates and UBP-1 allelic exchange transfections of *P. falciparum* parasites in culture should allow us to address these questions, and are planned. Transfection of mutated *ubp-1* genes in *P. chabaudi* would allow us to evaluate their role in an *in vivo* system. Up to now, however, successful stable transfection is not an established technique.

Previous studies have considered the ubiquitination/deubiquitination of *mdr1* in human cancer cell lines and its effects upon intracellular drug accumulation and responses to drugs. For example Zhang *et al*. (2004) report that in a human breast cancer cell line, the *mdr1* gene product, P-glycoprotein (P-gp), is constitutively ubiquitinated and that increased ubiquitination enhanced the rate of degradation of the *mdr1* gene product. Enhanced ubiquitination increased drug accumulation and sensitivity to the drugs doxorubicin and vinblastine, both of which are normally modulated by *mdr1* expression. Because the *P. falciparum* homologue, *pfmdr1*, has been shown to modulate the responses of malaria parasites to both artemisinin derivatives and to chloroquine, the effect of UBP-1 mutations upon possible post-translational modifications (e.g. ubiquitination or phosphorylation) of the *mdr1* gene product may be worthy of further study in *P. falciparum*.

We acknowledge that we have yet to provide direct evidence of a role for mutations in UBP-1 in artemisinin resistance in the rodent malaria parasite *P. chabaudi*. Nevertheless, we believe that the data presented here are important for two main reasons. First, they suggest new opportunities for genetic and biochemical investigations into drug resistance in malaria, especially in the vital area of resistance to artemisinin based therapies. Second, we believe that the demonstration of mutations in UBP and its deep involvement in central cellular processes such as responses to oxidative damage may encourage conceptual developments in our understanding of drug resistance, its genetic basis and its evolution.

## Experimental procedures

### Mice, parasites (cloned isolates, drug-resistant mutants), mosquitoes

Four- to six-week-old female CBA-inbred mice were used for all parasite infections. They had permanent access to 41B mouse maintenance diet (Harlon, UK) and drinking water supplemented with 0.05% p-amino-benzoic acid (pABA).

The *Plasmodium chabaudi chabaudi* (referred to here as *P. chabaudi*) parasites investigated here (Fig. 1) include the drug-resistant parasites AS-15CQ, AS-30CQ (Padua, 1981), AS-ART and AS-ATN (Afonso *et al*., 2006), which are derived from the drug-sensitive cloned isolate AS obtained from thicket rats, *Thamnomys rutilans*, in the Central African Republic (Landau, 1965; Landau and Chabaud, 1965). AJ is a genetically different drug-sensitive cloned isolate. All parasite clones were available as cryopreserved stabilates, deep-frozen in liquid nitrogen.

*Anopheles stephensi* mosquitoes were maintained in a dedicated insectary as previously described (Martinelli *et al*., 2005a).

### *In vitro* selection of artemisinin-resistant parasite lines and antimalarial drug assays

*Plasmodium falciparum* NF54 (chloroquine-sensitive) and 7G8 (chloroquine-resistant) strains were used for the selection of artemisinin (ART) resistance. Drug resistance selection experiments were started with approximately 5 × 10^10^ mixed stage parasites, which were exposed to 10 nM of ART for 10 days. Cultures were maintained carefully by feeding two to three times daily with ART-containing complete media. Cultures were smeared every 2–3 days and healthy asexual stage parasites were observed by examining Giemsa-stained thin blood smears. After initial drug exposure, ART drug concentration was increased to 25 nM for both parasite lines and kept at this level for the following 12 days. At this drug concentration dying asexual- and gametocyte-stage parasites were observed by microscopic examination of Giemsa-stained smears. The ART drug selection level was then increased to 50 nM that apparently eliminated asexual stage parasites, and cultures were further maintained at this drug level for following 4 weeks. During the entire selection process 30–40% red blood cells were replaced with freshly washed cells once a week. For both parasite lines, ring stage parasites growing in the presence of ART at 50 nM concentration were observed by day 45. Once the parasitaemias reached 2–3%, frozen stocks of ART-selected parasites were prepared using Glycerolyte 81. Parasites were phenotypically characterized for their ART response profiles using [^3^H]-hypoxanthine incorporation assays as described previously (Desjardins *et al*., 1979).

### Genetic cross

For each genetic cross, six mosquito cages (approximately 20 cm cubes) were set up each containing approximately 200 female *A. stephensi* mosquitoes, 5–7 days old. Glucose was removed from cages 24 h before mosquito feeds. Four mice were inoculated with both AJ and AS-ART (1 × 10^6^ parasites of each) and two mice were inoculated with either one of the parental clones. All mice were monitored for the presence of gametocytes. On day 6 post infection, mice with mixed infections or single infections of the parental parasites were each laid on top of a mosquito cage for 30 min. Glucose and water solution were then replaced and non-blood fed mosquitoes removed. After 7–9 days, 10 mosquitoes from each cage were removed and their midguts examined for the presence of oocysts. After 14 days following feeding, the salivary glands were removed from the mosquitoes and placed in 50% Ringer's solution and 50% heat-inactivated calf serum. The glands were gently disrupted using a pestle and mortar to release the sporozoites. This suspension was kept on ice, and injected intraperitoneally into a group of mice in 0.1 ml aliquots.

### Drugs and selection of cross-progeny (LGS)

When the sporozoite-induced infections (‘unpassaged’) reached parasitaemias of between 10% and 15%, the parasites were harvested, pooled and inoculated (1 × 10^7^ parasites) into two groups of mice, one treated with artemisinin (‘treated’) and the other left untreated (‘untreated’). Artemisinin was dissolved in dimethylsulphoxide and administered orally at a dose of 25 mg kg^−1^ of mouse body weight daily at 24 h intervals for 5 days, starting 3 h after parasite challenge. The treated and the untreated blood-stage cross progeny were allowed to grow until parasitaemias reached 30%−40% for untreated, and 15%−20% for treated, when parasites were harvested (days 6 and 12 pi respectively).

### Parasite harvesting

Mouse lymphocytes and other nucleated cells were removed from blood by filtration (twice) through 5 ml columns of powdered cellulose (CF11, Sigma) washed with citrate saline. Blood was then further filtered through Plasmodipur™ filters (Euro-Diagnostica) twice. The filtrate was centrifuged for 5 min at 3000 r.p.m. and the supernatant removed, leaving a pellet of packed cells. The pellet was resuspended in two volumes of 0.15% saponin in phosphate-buffered saline (PBS). After lysis of erythrocytes, PBS was added in excess to prevent parasite lysis. This solution was then centrifuged at 4000 r.p.m. for 5 min and washed twice in PBS. Supernatant was discarded and pellets stored at −70°C.

### DNA extraction, AFLP amplification, analysis and nomenclature

DNA from ‘unpassaged’‘treated’ and ‘untreated’ groups was extracted, prepared for AFLP analysis, and amplified using ‘non-selective’ and ‘selective’ oligonucleotide primers, as previously described (Grech *et al*., 2002). Polymorphic markers (between AS-ART and AJ) were named to denote specificity of the polymorphic band, the size of the band (relative to other polymorphic bands in the same gel lane) and the selective bases used. Thus, AJAG02CT denotes the second largest AJ-specific band obtained using *Eco*RI primers with AG as additional ‘selective’ 3′-nucleotides, and CT as ‘selective’ nucleotides on the *Mse*I primers. Genomic DNA from *P. falciparum* drug-selected and parental lines was prepared using DNeasy Blood and Tissue Kit (Qiagen).

### Determination of CIs of AJ-specific bands

Marker band intensities were measured with PhosphorImager and IMAGEQUANT software (Molecular Dynamics), and the values converted to relative intensity indices (RIIs) which estimate the intensity of a polymorphic marker in a mixture, relative to its intensity in the parental clone (Martinelli *et al*., 2004). CIs of polymorphic markers are defined as the RII of an AFLP marker in the cross progeny selected in ‘treated’ mice (RII_t_), divided by the RII of the marker of the cross progeny grown in a parallel ‘untreated’ group of mice (RII_ut_), and expressed as a percentage, i.e. CI = (RII_t_/RII_ut_) × 100.

### Mapping of AFLP markers

Markers with low CIs were identified and their position on the genetic linkage map (Martinelli *et al*., 2005b) noted. AFLP bands that appeared to be under selection were sequenced as previously described (Hunt *et al*., 2004b). The positions of corresponding sequences in the *P. falciparum* genome were determined as previously described (Hunt *et al*., 2004b). Briefly, *P. chabaudi* contig sequences containing highly similar AFLP sequences were identified by blast search. These contigs (usually containing a number of genes) were used to identify loci in *P. falciparum* containing orthologous genes, by blast search. These loci were expressed in the form pfxx-yyyy, where xx denotes the *P. falciparum* chromosome and where yyyy denotes the position in kb. The corresponding loci in *P. chabaudi* were predicted using a genome-wide syntenic map (Kooij *et al*., 2006).

### Sequencing, sequence analysis and SNP identification

Polymerase chain reactions were sequenced using the ABI PRISM Big DyeTM Terminator Cycle Sequencing Ready Reaction Kit (PE Applied Biosystems) according to manufacturer's instructions. Sequencing reaction products were purified by precipitation with sodium acetate and 95% ethanol, and washed in 70% ethanol, before analysis on an ABI 3700 sequencer. All fragments were sequenced in both forward and reverse directions. Sequencing results were analysed using SeqED v1.0.3 software (Applied Biosystems, 1992) and CHROMAS 2.31 (Technelysium Pty). To determine the sequence at the *pfcrt* polymorphic position 76, a 600 bp fragment was amplified by using the primers CF5C and BB84. The 5′ region of the *pfmdr1* gene encompassing the polymorphic site 86 was amplified as a 1.0 kb fragment using primers P285 and P423. For sequencing of the *pfmdr1* polymorphic positions 1034, 1042 and 1246, a 2.0 kb fragment was PCR-amplified with primers P213 and P215. The full-length 4.0 kb PfUBP-1 gene was PCR-amplified with primers P1595 and P1597 and fully sequenced on both strands using internal primers.

### Oligonucleotide primers

Oligonucleotide primers used for amplification and sequencing of candidate genes, and for pyrosequencing analysis are detailed in *Supplementary material*.

### Pyrosequencing

Assays to estimate proportions of SNPs were designed using Assay design software v1.0.6 (Biotage AB, Sweden). This defines two oligonucleotides as PCR primers, one of which is biotinylated, and a sequencing primer close to the SNP. The primers used for each of the assays are given in *Supplementary material*. Pyrosequencing assays were performed on a Biotage Pyrosequencer HS96A, according to manufacturer's procedures.

### Identification of DUB structural homologue and structural analysis

The sequence for *P. chabaudi* was entered into Phyre (Protein Homology/analogy Recognition Engine) software, which identified the catalytic core domain of HAUSP as a template (pdb id 1NBF) and created an initial protein model. The program Coot (Emsley and Cowtan, 2004) was used to manually examine the resultant model and adjust side chains to minimize steric clashes. The modelled mutations were created using Coot and also manually adjusted to minimize steric clashes. The structural figures were created using the program PyMOL (Delano, 2002).

## Supplementary material

The following supplementary material is available for this article:

Fig. S1 Pyrosequencing data at 14 loci (chromosomes 2, 6, 8 and 14) in the 3 individual crosses (AS-ART × AJ) which were components of the pooled crosses analysed by genome-wide pyrosequencing in the main text of the paper.

Table S1 Primers for amplifying and sequencing candidate genes in *P. chabaudi*.

Table S2 Primers for amplifying and sequencing candidate genes in *P. falciparum*.

Table S3 Primers for genome-wide pyrosequencing (amplification and sequencing).

This material is available as part of the online article from:

http://www.blackwell-synergy.com/doi/abs/10.1111/j.1365-2958.2007.05753.x

(This link will take you to the article abstract).
