# Supplementary material for: Gene encoding a deubiquitinating enzyme is mutated in artesunate- and chloroquine-resistant rodent malaria parasites
Source: Mol Microbiol. 2007 Jul 1;65(1):27–40. doi: 10.1111/j.1365-2958.2007.05753.x (PMC1974797; doi:10.1111/j.1365-2958.2007.05753.x)
Supplement: Fig. S1 — Pyrosequencing data at 14 loci (chromosomes 2, 6, 8 and 14) in the 3 individual crosses (AS-ART × AJ) which were components of the pooled crosses analysed by genome-wide pyrosequencing in the main text of the paper. [file mmi0065-0027-S1.pdf]

Supplementary Material

Gene encoding a de-ubiquitinating enzyme is mutated in artemisinin- and chloroquine-resistant rodent malaria parasites.

Paul Hunt[1], Ana Afonso[2], Alison Creasey[1], Richard Culleton[1, 6], Amar Bir Singh Sidhu[3, 7], John Logan[1], Stephanie G. Valderramos[3, 7], Iain McNae[4], Sandra Cheesman[1], Virgilio do Rosario[2], Richard Carter[1], David A. Fidock[3, 7] and Pedro Cravo[5]

[1] Institute for Immunology and Infection Research, School of Biological Sciences, University of Edinburgh, Ashworth Laboratory, Kings Buildings, Edinburgh, EH9 3JT, UK. [2] Centro de Malaria e Outras Doenças Tropicais/IHMT/UEI Malaria, Rua da Junqueira 96, 1349-008 Lisbon, Portugal. [3] Department of Microbiology and Immunology, Albert Einstein College of Medicine, Forchheimer 403, 1300 Morris Park Avenue, Bronx, NY 10461, USA. [4] Institute for Structural Biology, School of Biological Sciences, University of Edinburgh, Swann Building, Kings Buildings, Edinburgh, EH9 3JR, UK. [5] Centro de Malaria e Outras Doenças Tropicais/IHMT/UEI Biologia Molecular, Rua da Junqueira 96, 1349-008 Lisbon, Portugal.

Running title: Deubiquitination mutations and drug-resistant malaria

Key words*: Plasmodium*; artemisinin; chloroquine; ubiquitin; mutation; drug resistance

Accession numbers
EF100417 – EF100437 inclusive

Corresponding author
Paul Hunt
Institute for Immunology and Infection Research
University of Edinburgh
Ashworth Laboratory
Kings Buildings
Edinburgh
EH9 3 JT
UK

Telephone
+ 44 (0)131 651 3601
Fax
+44 (0)131 651 3605
Email
Paul.Hunt@ed.ac.uk

This supplementary material includes

Table S1. Primers for amplifying and sequencing candidate genes in *P. chabaudi* (1. outer primers; 2. inner primers)

Table S2. Primers for amplifying and sequencing candidate genes in *P. falciparum*

Table S3. Primers for genome-wide pyrosequencing (amplification and sequencing)

Figure S1 A-C. Pyrosequencing data at 14 loci (chromosomes 2, 6, 8 and 14) in the 3 individual crosses (AS-ART x AJ) which were components of the pooled crosses analysed by genome-wide pyrosequencing in the main text of the paper

Table S1. Primers used for sequencing of *P. chabaudi* candidate genes

1. Outer primers

P. falciparum				*P. chabaudi* outer			
				Forward		Reverse	
Gene name	Gene ID	approximate position	contig id	name	sequence	name	sequence
vacuolar ATPase subunit C	MAL1P1.52			Pcvas-1F	GTTTGGAAATTGGGAACAAGAG	Pcvas-2R	CGGATTCATTAACAACTTGTGG
				Pcvas-3F	GCGTTTTAGTTGGGATGATG	Pcvas-4R	GCAATAAATACATCTGAAAAAGCAG
				Pcvas-5F	GATGGAAATACATTATGGAAAGC	Pcvas-6R	CGCGATAGTGCGCTTTTATTTC
lytb	MAL1P1.35		929.1	pclytB-1F	GTATGGGATGCTAAATGTGCG	pclytB-2R	ACTAATGGGCATGTTGCATCG
				pclytB-3F	GAATGAAGTACCAGATGGTAG	pclytB-4R	GCATAACATATAGAGCCACTTG
				pclytB-5F	ACATTAAGTATAGATGATTGCTC	pclytB-6R	ATGTGTAAATAGGATGCTTTCC
ubp-1	MAL1P1.34b			PcUb-1F	CGCCCACTCTCACATTGACA	PcUb-2R	CTGGTGTTGGCAAATCAGAATC
				PcUb-3F	CAAGCTTCTCTCACAGCATATC	PcUb-4R	CCCATTATCTTGTTCTTCTTTAC
				PcUb-5F	CCGGACTATCCTACTTCTTTG	PcUb-6R	AGTTTTCATATTCATCTGGTCTC
				PcUb-7F	CGTAGAACAATTCAAACCAATGG	PcUb-8R	TTGATGGGCATTTCGTTAGAG
				PcUb-9F	CATGTTCCTGCAAAGTCAAG	PcUb-10R	TAGTAATAGCTGAGTCGTCC
				PcUb-11F	CACAGTGGCGTGTCAGCATC	PcUb-12R	GTTTTCGTTCAAAGGTGCACG
ubp-2	MAL7P1.147			pcubp-1f	CAGTGCGACATACAACAAATAG	pcubp-1r	TTGTACATTACTAAAACCCCAATCA
				pcubp-2f	AAGAGGTGTAGTAGATAATGAAA	pcubp-2r	CAAATTGGATAGGGTGTTCAG
				pcubp-3f	AGAATCATGGAGCTACATGC	pcubp-3r	CATCAAGCTGAGGGTTTTTATC
				pcubp-4f	GTTCTGAAAATGAATCCATGTC	pcubp-4r	TCTTTGCATACCATACCCATC
				pcubp-5f	GATACGCTCTTTTGGATGGA	pcubp-5r	ATTTGGTACTGAACAATGATGG
				pcubp-6f	GGAGGATATGATATAGAAATTG	pcubp-6r	GTAGTTGTAGTACATCCAGAA
				pcubp-7f	AACGATTCCATTCTTATGTCAC	pcubp-7r	CTAGATCCTGATACAGCAGTTC
				pcubp-8f	CATCAACAAATAGTGCATCTAAC	pcubp-8r	GATTAGGGTATGATCCATTATC
				pcubp-9f	GTTTAGATGTTATGCATTGTGATA	pcubp-9r	GTATTGTGTAAAATAGCTTCAGGA
				pcubp-10f	CAGAAATATCTAAATCGACTCTA	pcubp-10r	AATTCTTCTTCAACATTGTCTAGA
				pcubp-11f	GCTGACTGTAATAATAAAATGGC	pcubp-11r	CAATCCTGTTTGCCTTCTTAC
				pcubp-12f	GATCAATACAAAATTACAACAGACG	pcubp-12r	TTTCATCTGTATAACTAGGATCA
				pcubp-13f	CGAAATTGTTCAGGGGTTATA	pcubp-13r	TCAGTTGGTTCATTTCGAGC
				pcubp-14f	CGCATGTACAATCAGTTTCTGC	pcubp-14r	GTTAGATCTCCAAATTCTTTGCC
				pcubp-15f	CGAACCATTGATACATCTGG	pcubp-15r	GCTATTTTCATTCCTATCTGTGG
				pcubp-16f	ATTCATGTTTTTAACCAAACCCC	pcubp-16r	AGCCGTTTTCCATTGTTTTATCC

2. Inner primers

P. falciparum				*P. chabaudi* inner			
				Forward		Reverse	
Gene name	Gene ID	approximate position	contig id	name	sequence	name	sequence
ubp-2	MAL7P1.147			pcubp-1fi	TTGCATAGGCAGGAAGAAATAAAC	pcubp-1ri	TAAATGCTGATGCCACTGGA
				pcubp-2fi	ATATTCGCAATGGAAAATTTGTCC	pcubp-2ri	CGATGTTTCCATGCTTGATATG
				pcubp-3fi	TCGTAAGTCTGTTTGTATGATGA	pcubp-3ri	CATTGGAGTTATGCATAGAGC
				pcubp-4fi	GTTTCACACATCTGTTCTTCTG	pcubp-4ri	TTCTACTTCGCCTTCAAACATC
				pcubp-5fi	CGAGTGATGTCTTTACCCAAC	pcubp-5ri	AGTTTACATCTTCGGACAACC
				pcubp-6fi	CGATAGGATTAAACAAAGGATG	pcubp-6ri	GTGTCTTCATCATTAAATCGGTA
				pcubp-7fi	ATACTACCGATTTATCTAACTCG	pcubp-7ri	TGTATCATTCACAGGGAGTAG
				pcubp-8fi	CTACTACATCACCTTCCGC	pcubp-8ri	AGTTAAATATTAGGAGATCACTAG
				pcubp-9fi	GGATGATAAATAAGATACTAAAAGC	pcubp-9ri	AGCTAAATTCTGATCAATCAAGC
				pcubp-10fi	CCTACACAAAACTATGATCTAACC	pcubp-10ri	ATTGTATGAGAACCCAAATTGGC
				pcubp-11fi	AGCTCACAACCTACTATTGATG	pcubp-11ri	GGTCACCAATCTGTTCATTTAC
				pcubp-12fi	ATCAAAATACTGAATACCCTCAC	pcubp-12ri	ATTGGACTATTGAGGGGAGG
				pcubp-13fi	GATATTAGATGCCCAACAAAACA	pcubp-13ri	ATATTCATTGTTTGCTAAGGTGC
				pcubp-14fi	CCATAGGTAATGATAATGACTC	pcubp-14ri	CAAAACTGTATCCGAAAACTTC
				pcubp-15fi	GTAACCCCACTCCGAATGG	pcubp-15ri	GTTAGATCTCCAAATTCTTTGCC
				pcubp-16fi	ATACGAAAGAGAACAATTTTACCG	pcubp-16ri	AGTGAAACAAAGTGGAATGAAATC

Table S2. Sequencing primers used for sequencing of *P. falciparum* candidate genes

P. falciparum ***P. falciparum***

Gene name	Gene ID	Forward		Reverse	
		name	sequence	name	sequence
crt		cf5c	AATTCAAGCAAAAATGACGAGCG	BB84	GACTGAACAGGCATCTAACATGG
mdr1		P285	ACTATTTGTTGCTTCTAAAGCTTTCAT	P423	AAAGATGGTAACCTCAGTATCAAAGAAGAG
		P213	GATACAGGTAATGATGCAGATAA	P215	TACGTATTTTTTATATATTCCATCTTGTGC
ubp-1	MAL1P1.34b	P1595	ATGCCTATCCCCAGCATGTCGA	P1597	AAAAATTAACCTGAATGGATAA

Table S3. Primers used for genome-wide pyrosequencing

PYROSEQUENCING PRIMERS (bold indicates the biotinylated primer)

***P. falciparum* locus**		***P. chabaudi* sequence**		
Gene ID	locus designation (approximate position)	Amplification		Sequencing Primer
		Forward	Reverse	
**CHROMOSOME 1**				
MAL6P1.24	pf 06 0142	TCGTCTAATGCGCTTCTATCTTT	**AAAAACCAAGCATGCCACAT**	TCTATCTTTCAGTGCGTTA
MAL6P1.68	pf 06 0317	**TTGCGGATTTGATGTCGTATAA**	AGCAATAAAAACGTTGCATAACAG	ACAGAAATATATATGATGGA
MAL6P1.101	pf 06 0472	ACGATGGAGGCATAGGTATATTCA	**CACTGTTCGGCTTGTAATAGATGT**	TGCATAAAAAATCTTCTGT
MAL6P1.290	pf 06 0600	**TGAATTTAGCATAATCATAATTTG**	TGCTTTACAAAGTTCAGAATGTTT	AGTTCAGAATGTTTATCTAA
**CHROMOSOME 2**				
MAL1P2.32	pf 01 - 0406	AGGCGCGTCAGTCAAATT	**ACTATTTCTTCTGCGGCTCCTA**	ATTGGAGGGTTCTAAAGAG
MAL1P2.31	pf 01 - 0399	AAGGGTAACAAGGTTGAAGATGGT	**GTTGTGATAATCGTCGTTTAATGC**	CAAGGTTGAAGATGGTG
MAL1P2.06	pf 01 - 0323	ACGTCTTTCTATGCAGACACACA	**TTTCTTCCCCTTCATAAAAATCG**	CTATGCAGACACACAAGTAG
MAL1P1.54	pf 01 - 0265	**TAGCTGTTGCTGCAATTCCTGAA**	TTGACCATTCGTCTGGTTCCTAAA	TCTGGTTCCTAAAGCTAA
MAL1P1.34b	pf 01 - 0197	**TTGAACAATTAGGAGGATCAGAA**	CGAGCATTTGTATTTATTGTTTCC	TGCAGGAACATGAAATG
MAL1P1.74b	pf 01 - 0150	**GAATTCGGATATTTTCGATAGTTG**	TGAAAAGGGCCATAAATCATACAT	GCCATAAATCATACATCCA
MAL1P1.20	pf 01 - 0129	GGGAGCTAATGCTTTTCAAATAAA	**CCCGAGTTGATACACATCTGTTTA**	AATTATTTTAATCAAAATGG
MAL7P1.145	pf 07 - 1006	TGACTCCTGATAAAAGGGAAGTTT	**CAAGATGTGTTGTTTTGGGAGTTA**	TACTTTTGAAAATGAATTGT
PF07_0121	pf 07 - 1151	CGGAATCAAATTATAGCGAATTGC	**ATCTCCTGGAAATAAATGTCTTGC**	AGTAAAATTAGAAAAACT
**CHROMOSOME 3**				
PFB0130w	pf 02 0139	**CGAGCTAATAAAAAGTCACCAGAT**	TGGGCTCTCTTTTACATGATGAT	TGAAAAAAGACGAGGATC
PFB0305c	pf 02 0278	TGCCGACCCAAAAGTTATCCT	**TTCCGCAAAAACTGTAATTCACTG**	CATGGTACTAATTGTATCGC
NP_473027	pf 02 0452	CAGAATGAAAAATAAAAATGCAAT	**TGTCTATAGTCTTATCATGCGATA**	TGAAAAATAAAAATGCAATA

**CHROMOSOME 4**							
MAL3P3.17	pf	03		0419	**CCTTTTAAAATCGAAAAGAAGTGA**	CCGTTGCTTTATATACACAACCA	TTGCTTTATATACACAACCA
MAL3P5.2	pf	03		0560	ATTTTCCTCATCAATCGAAACAT	**GGAATGTGGGGACTAGCAAAA**	GTTTAGGCACTAAGACAGAC
PFI0245c	pf	09		0230	AACTGCGATGTAAGATATGTCCC	**ATCAACCAATTAAAAGGTATTCCA**	ATTAGAAATAAATTATGAAA
PFI0160w	pf	09		0141	AATATTTGAATCTGCACCATACCA	**TTGCACAAAAGGCTATTGTTCTC**	CACCATACCATGTTTTTC
**CHROMOSOME 5**							
PF10_0209	pf	10	-	0868	AGGTACCATATATTTCCAGATTTA	**CCCGTTTTAATATTTACAGATAGT**	TCTTTTGATTTATCAGAAGT
PF10_0242	pf	10	-	1036	CATTCGCTCCCGAAAATACAA	**TCAGTGTTTGATGTTGCTCTAAGA**	GCTGCACCTTCTACACA
PF10_0287	pf	10	-	1203	**GACGAACACAGTTTTACAACAATG**	CTTCGACCATCATGAATAGAATGT	ATATGATTCTGATAGTAAAT
PFD0985wa	pf	04	-	0942	TGGACCATCTGGTGATAACAAG	**GACCCATCACTAACATCACTTGTA**	TAAAGGAAATGGAAATTTA
PFD1065c	pf	04		1072	**CGTTTACCATACTTCCTAAACACA**	TCAAAACTTAGCGATGCTGTCTAT	TTTTGGGTATAATATTTCTC
**CHROMOSOME 6**							
PFL0080c	pf	12		0102	**ATTAGCAGAAAAGGGAGAAGACAA**	TGGTATATCACAAAAAGGATCAAA	TGATTTATTTCAAGTGGG
PFL0290w	pf	12		0259	**AGAAAGTATGAAACAACTTGGATT**	CTTCATTGGAAACATTATTTTGAC	ACATTATTTTGACAATTAGG
PFL0420w	pf	12		0390	**TGGTGGTTTACTTCTCGTGCTATT**	AATGAAGCGGATTTTTTTGAGA	ATGCGTATGAAAAAATTAA
PFL0545	pf	12		0484	AGTAGCAAAAGCAGAGCAGAGAC	**GCTCTTCCAGCTCTTTCATTACC**	AATCAAGTATTGAGTAATGG
PF07_0105	pf	07		0962	**TGGAAAAAATAAGATACCCCCAAAT**	TGTTTGAAAAAACTTCCAAATGAT	TGATTGATGTATTGGGAC
**CHROMOSOME 7**							
PF11_0395	pf	11		1498	TTCTCCGTTTACTAGCGATGTTT	**TTCCCTCTTTCCAAATATTATCAG**	AGTTAAATCCATCCATTTC
MAL8P1.58	pf	08		0431	TTGGAGGGAATATGATTATTAGTT	**CATAGCGATAAAGAAATGGAAAAA**	TGCCTATATTGTGTAAATGA
MAL8P1.78	pf	08		0580	CCTCACAATATCCTTGATGACACT	**TGTTTGTCCAGCCATAAAAGC**	TTGTTCATTACTCTTGAATT
PFD0725c	pf	04		0675	CATCTTGAGAAGTTAATGGGAAAA	**TTTCAAAACCCTTTAAAAACAACA**	GTTAATGGGAAAACAAC
PFD0875c	pf	04		0820	**TTGGAAATTTAAAAGCGAGACATC**	CCGCCATAACTTCATTTAATTGAT	ATTCATAATTCAGTATACTA
**CHROMOSOME 8**							
PF07_0016	pf	07		0175	AGCATTTTCATTTGATGTGTCTTT	**AACCAATTAACCACCTATGACAAA**	TTATTATCATCTTCATCAGT
MAL3P6.13	pf	03		0719	**TTCCGCTATAAAAAAACGAAGAAC**	CTTTTTAATGCATTTTCACTACCT	TCACTATTTAAATCTTTCAT
PFI0685w	pf	09		0601	**TGTTGTAGATAAGACGAGTGGATT**	CTATGTTTTTTCCAAACCATCTTT	AACTTTTTATCCACAACATT
PFI0925w	pf	09		0777	GGATGCTTCCAATGAAAATGTAG	**GATTTATTCCAAATGAGATGACCA**	ATTTAATGAAATATATAAAG
PFI0950w	pf	09		0795	AACCTGGAGATGTCGATAAATATG	**TTTCAATTCGAGATTCTTCATCAT**	TTTGTTCATTTTGGTGAG
PFI1020c	pf	09		0852	TTTTAGATGCATGAGGAAAGATT	**AATGGTAACAGGCAAATATCCAA**	ATGCATGAGGAAAGATT
PFI1090w	pf	09		0903	ATATTTGATCGCAAATTTTATCGG	**ACAAAATTAAAAAACAATGGACCA**	GGATTCCGAAGTAAATAAAA
PFI1140w	pf	09		0944	**GAAAAGCGAAACAAATCAACATAC**	TCCAGTAAGACTTGTGGCTATAGG	GCTATAGGCTTATTAAATTG
MSP-1	pf	09		1203	CGAATTAATGCACGCAATAAA	**TTTCACAATAGTATTTGCACACA**	CACGCAATAAATTTTTACTA
PFI1700c	pf	09		1370	CCCGTTTTTTTATTGTCAAACA	**GGAAGAATGTCAGCGCTATTTAA**	TTTTATTGTCAAACACGTT

**CHROMOSOME 9**						
PF11_0065	pf	11	0234	GGATGGATATATGGTATACTTCGA	**TGATGTCAAAGGTCGATTTGTT**	GTATACTTCGACCATCATG
PF11_0177	pf	11	0651	**GAAGCAGGTTTGGAAGCAGAAAT**	GTTGCACATGCATTCGGAATAAC	CGATCATATGTTTTGCC
PF11_0225	pf	11	0820	**CTATTGGTTTTGCATTTGAATCTT**	GATGAAACACCATTTAAGTTTTCT	AATGCAAATATTATAATAGC
PF11_0294	pf	11	1100	AGCAGGAATAGCTGTCGAAAATA	**GGTACAATGAGCTGCTCCAATTT**	AATTATGTAACATCCGTTAG
PF08_0010	pf	08	0126	GGCCCATACCATATTATGATTACA	**TAAAAACACCTGGAAATGCTGATA**	ACAAAAATGGATTCAAAA
**CHROMOSOME 10**						
PFD0285c	pf	04	0290	GACAAATTGCACATTTGATGG	**GCAAACCATGCTTCATCAAATAAC**	ATTGCACATTTGATGG
PFD0350w	pf	04	0370	GGGGTTGTATTAGAAATAGGAGCA	**CAGCTGTTCACACATTTTTTCACT**	GAAATAGGAGCAGGGAC
PF14_0244	pf	14	1029	**TTTAGATCAGTAGGTTTACCTTGG**	CATGCAAAAAATGTCCAGATTC	TTACATCAACCTAGTAGTCA
PF14_0139	pf	14	0562	TCGCCCTATATGACGGACTTTGA	**CAGGTGATCGTTTTGTACGCAATA**	TCAGTTTGTAAGCTAAAAAT
PF14_0063	pf	14	0240	**GGTATGATGAACTTCAACCAGAAA**	CCCTTCTAATTGATCTAGCTCCAA	TCTCTCTTAAGAAATTGACG
**CHROMOSOME 11**						
MSP-8	pf	05	0115	**GTGATCTTTAGCTGCTTGATCTTC**	GGAGAATACGATCTTAAAGCTATG	TTTAAAAGATGCTTTAACA
PFE0690c	pf	05	0585	**GCTGATGTTTCAATAAATGGTATG**	AGAGGAGCCGATGGAATAATAATA	ATAAATACACAAATGAAGAT
MAL6P1.255	pf	06	0782	ACTGGTTGAAAATTTATATGAAGA	**GTTTCATATTTTTGGAAAATCAAT**	AATATGTTTAATGCTTGTTC
MAL6P1.220	pf	06	0902	GTATGTACACCCATGTGGTGATTT	**TATTTCCATCAACCGAAACG**	TTAATCATATTAGTGGAACA
MAL6P1.193	pf	06	1001	TGCTTAGGCATATTTGCTTCAA	**TTGAAGGTTTTGTAGCTCCTTCGA**	CCATAAAATTATATTCTGG
MAL6P1.151	pf	06	1154	TCTTTTTTTTTCGAGGACGATA	**TGGAAAAGAGTTTTTAAAATATGC**	AATACTAAAAATTTAATACA
MAL6P1.125	pf	06	1305	GTGAACAGCTTTTAATGGCAGAA	**AAATCATCCCTATAAATCCCACAT**	CATTTGTTGGAGGGAA
MAL6P1.119	pf	06	1338	GTTGTTTGGGTTGCTGAAAATAA	**TGCAACACCAGGTAGATTATTAGA**	ATCCTATTTGAAGTAACCTA
PF13_0278	pf	13	2057	**AATAAAAGAAAAGGTTTTTGTGTT**	CATCTTTTCAGCAGAAAATTCATT	GCAGAAAATTCATTATTAAA
PF13_0357	pf	13	2620	**TCAAGTCGGATTTTTATGTAAAGG**	CAATTCTGCAAATTATTTAGAACA	CTGCAAATTATTTAGAACAA
**CHROMOSOME 12**						
PF10_0034	pf	10	0141	**ACATGCGTTGTATTTTCGTATGT**	TGGATGAAGAGGAATGCTTTACA	TGAATTTGTTAAATTCACTT
MAL3P7.12	pf	03	0868	GTCGCAAACACATCGAAATC	**CATCGGCAAGCACCATTT**	TCGTGTTTTTCATGATTT
MAL7P1.32	pf	07	0370	**CTTTGTGTAATGATCCATATTTCC**	GAGTTGGCACTTTTGTCAACTAA	AACTAAAAATCCACGAGGG
MAL8P1.134	pf	08	1006	**CGACCAAAACAAACAGTAACAGA**	TCCCCTATATGGATTAACTTGAAA	TCTACTTTGCCTGGACA
PF08_0130	pf	08	1156	**CAGCAGCCACTATATTTCCACTTA**	ATGAACGAGTCAGGTAAAATTGTG	AAAATATCGAAAAAAATTAA
PFE1010w	pf	05	0836	GGCTATTTAATCGAAAAACGTATT	**TTTTTTCTCCTTCCTTGCATAGTT**	TTTGAAAATACTGAATTTAT
PFE1560c	pf	05	1286	**AAAATTTTATGCATTTTTAATGGG**	CCCGTTTTAAAACAAAATATTTCC	TTTTAACAATTGTATGTTGG

**CHROMOSOME 13**						
PF14_0702	pf	14	2986	TCGACTAGTTCCACTTCAATTTTA	**TACACATGCATTGAAAACAATCC**	AAGAAATTCTGAAGGGC
PF14_0386	pf	14	1658	**GTTTGCGCTCAATTCATCTTTAT**	GGTATATCGAAAATGTTGTAGGTA	AATATCAGCAAACATATCAT
PF14_0416	pf	14	1794	**GTCGGTAGTGAGCATAAAAATGAA**	TCCTCTTTGATGTCTTCGATTTTC	TAGTTCTCTTTTTAAACATT
PF14_0571	pf	14	2445	**TTGAACAATTAGGAGGATCAGAA**	CGAGCATTTGTATTTATTGTTTCC	TGCAGGAACATGAAATG
PF14_0632	pf	14	2708	AGGCAACGAAGAAGCTGTTAATA	**TGGCATTTCGTCCACATATATCTA**	TGCCACTTTTAACTGATAC
PF13_0143	pf	13	1050	**AGATTTGCAGATGGAGAAGTATCA**	TTTTCTATCTTGACGTGCATAACC	GATTTTCATTAACTGGTGG
MAL13P1.172	pf	13	1356	TTGAAACTGCTTCAGATTTGTCTT	**TCCTTTCATTACCCAACTTATGC**	ATCATCTTTACATATGCTTG
MAL13P1.256	pf	13	1956	GTTCGCTTGTTTCTTTCCAATCA	**TTTGAGCCAGAAAACAGGAAGAG**	TCCAATCAGATTGAAAAT
**CHROMOSOME 14**						
MAL7P1.66	pf	07	0524	**TGACGGGAAAAAAATATGTCTG**	GGCCAATGGCTAGTGCAATA	CAATGGCTAGTGCAATAAT
PF07_0065	pf	07	0604	**TGTTTCGAACAATTT**	AATTGCGGGTATGTTGC	TGTTGCCTTTGATCAATA
MALP 8P1.92	pf	08	0650	TTTTGCATATACACAATTGCACAT	**CAAGTCTCAAAGCGCACAAATA**	TCGAGGCAGTAGTAAATG
PF08_0098	pf	08	0819	GAAAGCTATCCGCAACCTATTAGA	**TTCGACTGATGATTTTACAAGTCT**	TTATGTCTAATTGATTTTGG
PFL0735w	pf	12	0627	GGTGGACCATCTTCTTCTAAACTT	**TGCTTTATATAATCAAGGCCCTCA**	CCATACCTTTCAAAACAGT
PFL0925w	pf	12	0745	AGAAGAAAGCGATGGAATTTTAGA	**TTTTCTTTCAACATCTCGAAAACT**	TTAGAAAAATTAAAAGGTGA
PFL1120c	pf	12	0934	ATGCTGCTGCTATGAGATATACA	**AACATCAACAAGGTTATGACTTGG**	GAAAAAGAACCTAAAGTG
PFL1450c	pf	12	1242	**AATCGAAACCCTCCATATGCTAAT**	TGCAGGTTGTCATTAATTTTAGCT	CTTAACAAAAAAAAAGAGTC
PFL1940w	pf	12	1682	**TAACATCATTGCGATTAAATGAAA**	GGCAGATGAATAATACTTGTCGAT	TTCTGTTAATACTGGTTTTG
PFL2100w	pf	12	1830	**ATCTGCACTTTCCTGACTACTAGC**	AAGCAGAAGCCTTTATACACTGGT	TATGACAGTGGAAATCCT
PFL2505c	pf	12	2125	GAATGAAGGTGCTAATTTTGAAA	**TCGTTGCATTCACCTGATTT**	ATTAGAAAAGTTTTGGC

Figure S1 A-C. **Pyrosequencing analysis of individual AS-ART x AJ crosses (14 loci on chromosomes 2, 6, 8, 14)**

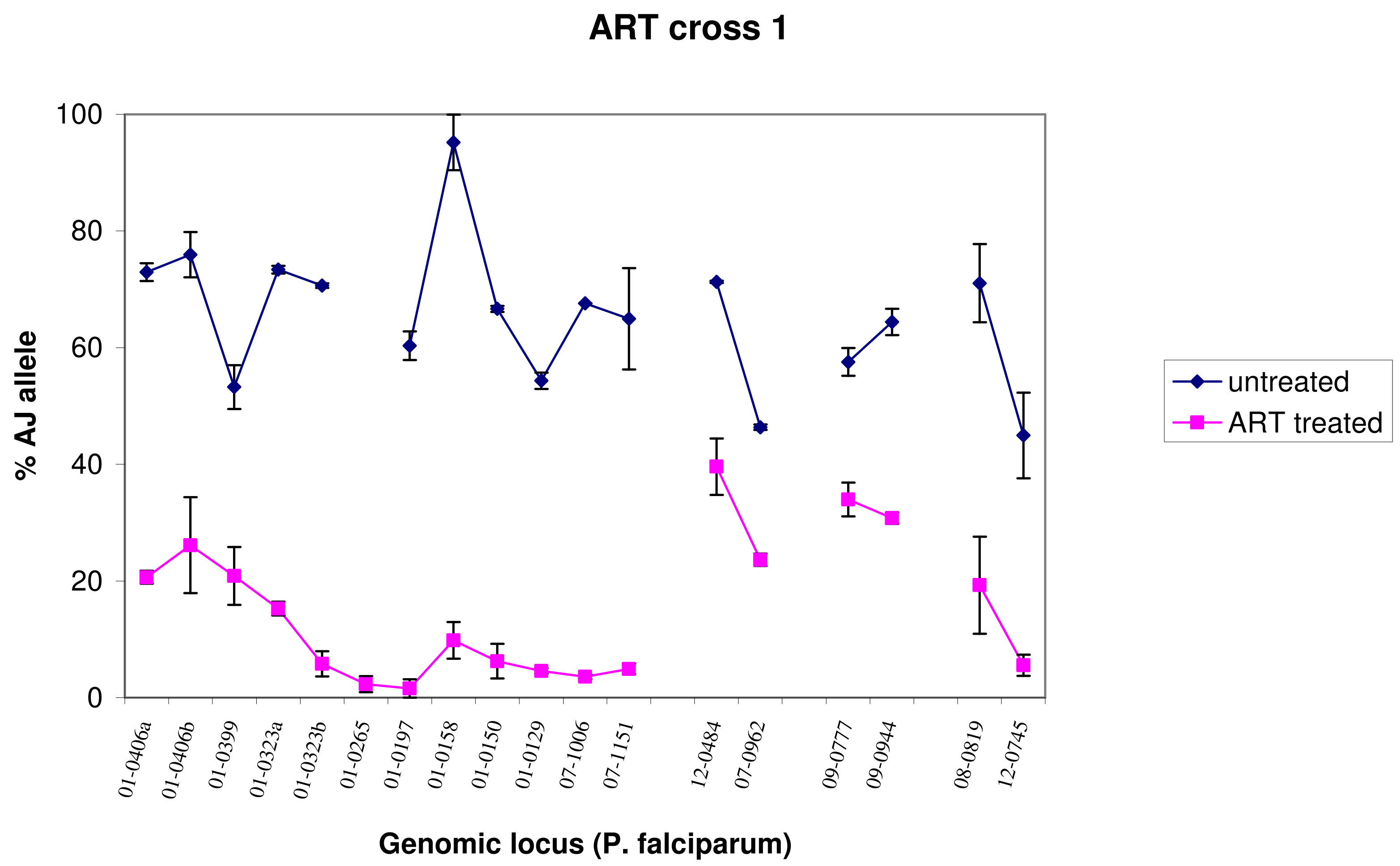

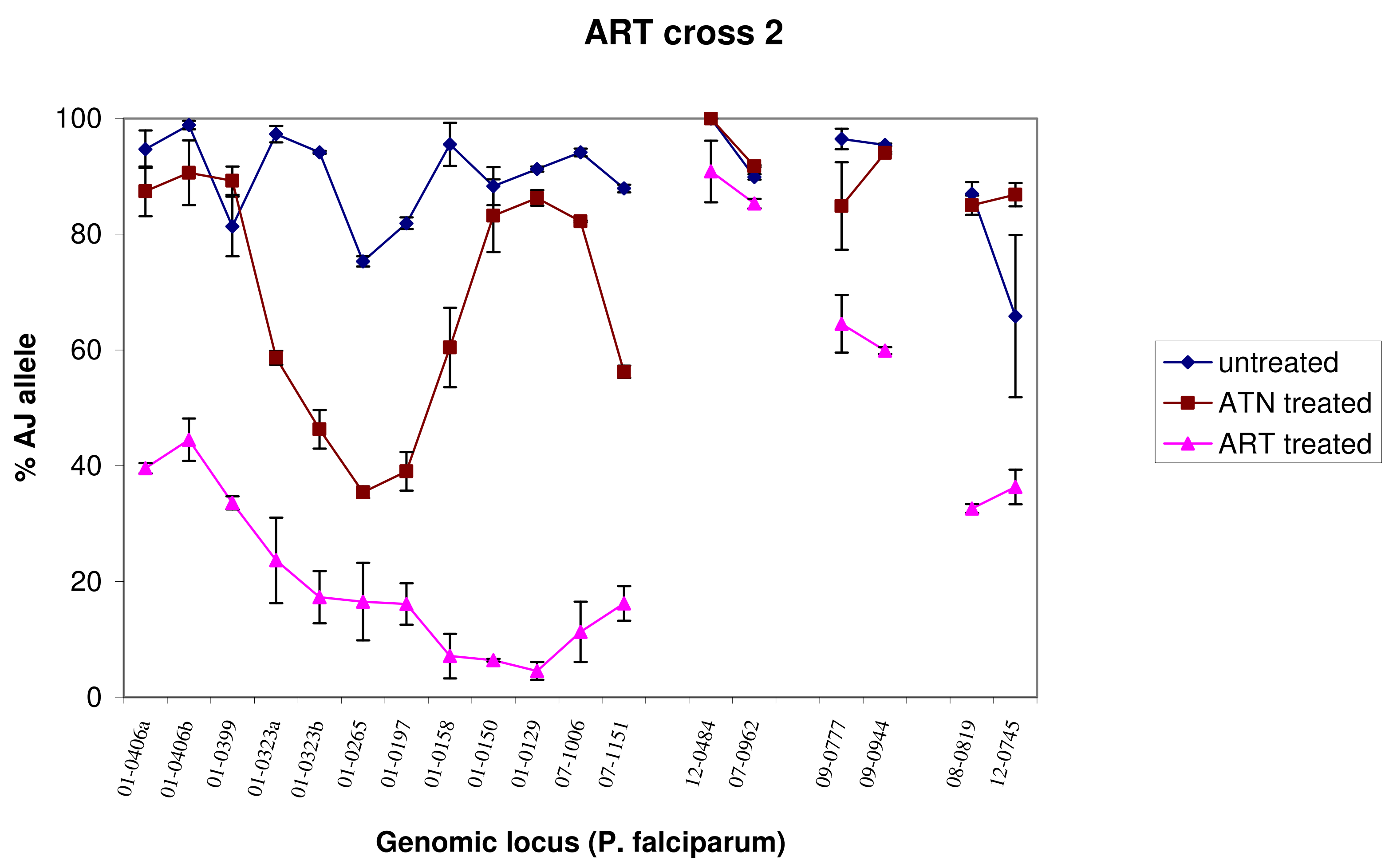
ART cross 2
% AJ allele
100
80
60
40
20
0
01-0406a
01-0406b
01-0399
01-0323a
01-0323b
01-0265
01-0197
01-0158
01-0150
01-0129
07-1006
07-1151
12-0484
07-0962
09-0777
09-0944
08-0819
12-0745
Genomic locus (P. falciparum)
untreated
ATN treated
ART treated

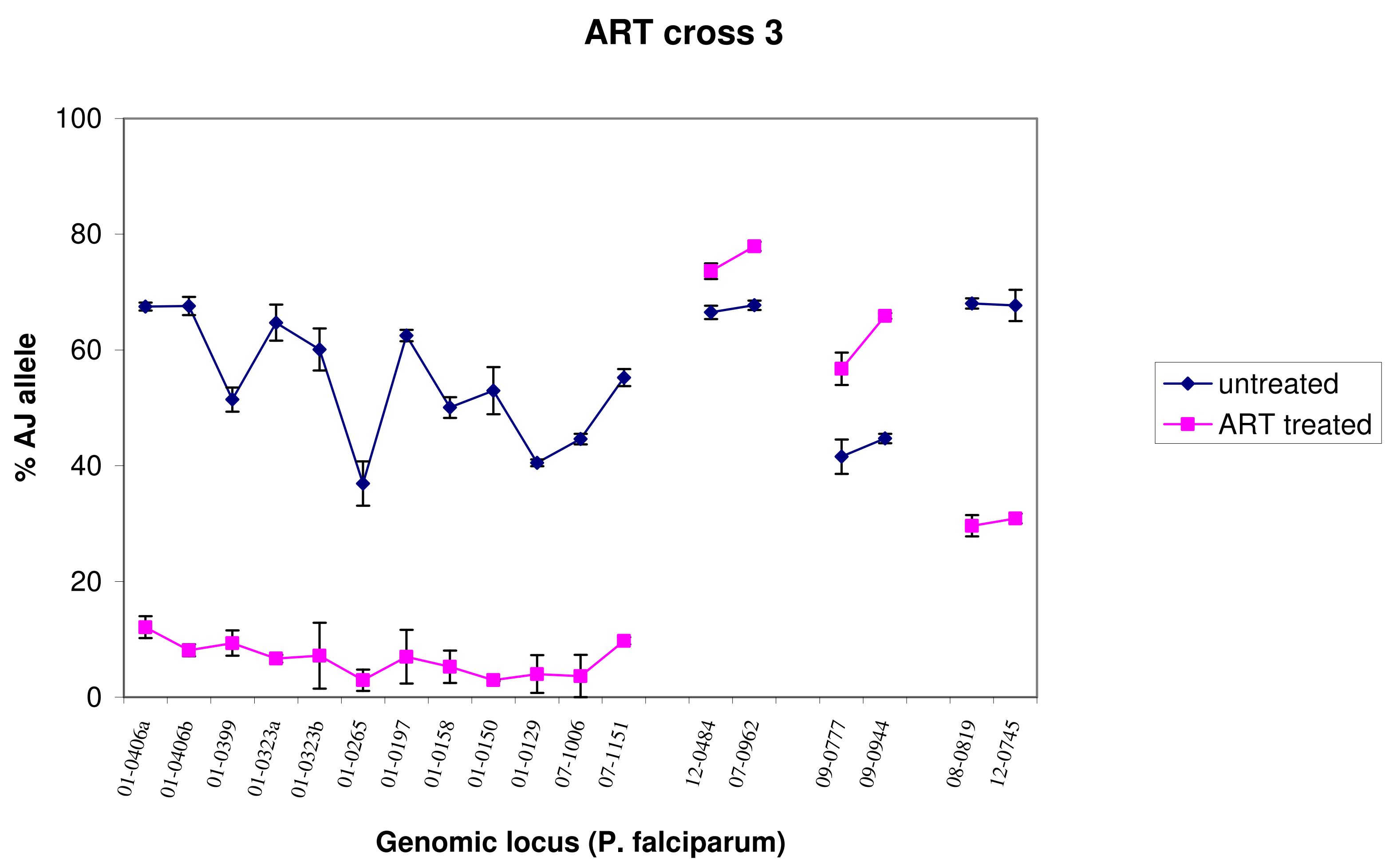
ART cross 3
% AJ allele
100
80
60
40
20
0
01-0406a
01-0406b
01-0399
01-0323a
01-0323b
01-0265
01-0197
01-0158
01-0150
01-0129
07-1006
07-1151
12-0484
07-0962
09-0777
09-0944
08-0819
12-0745
Genomic locus (P. falciparum)
untreated
ART treated
